# MicroRNA-545 suppresses progression of ovarian cancer through mediating PLK1 expression by a direct binding and an indirect regulation involving KDM4B-mediated demethylation

**DOI:** 10.1186/s12885-021-07830-8

**Published:** 2021-02-15

**Authors:** Hailing Zhang, Ke Zhang, Zhen Xu, Zhilong Chen, Qian Wang, Chenyang Wang, Jinquan Cui

**Affiliations:** Department of Obstetrics and Gynecology, The Second Affiliated Hospital of Zhengzhou University, No.2, Jingba Road, Zhengzhou, 450014 Henan People’s Republic of China

**Keywords:** microRNA-545, KDM4B, PLK1, Ovarian cancer

## Abstract

**Background:**

Ovarian cancer (OC) is a life-threatening gynecological malignancy where dysregulation of microRNAs (miRNAs) is frequently implicated. This study focuses on the function of miR-545 on OC development and the molecules involved.

**Methods:**

miR-545 expression in OC tissues and cell lines was determined, and its link to the survival of patients was analyzed. Altered expression of miR-545 was induced to determine its role in proliferation, apoptosis, migration and invasion of OC cells and the angiogenesis ability of human umbilical vein endothelial cells (HUVECs). The targeting mRNAs of miR-545 were predicted and validated through luciferase assays. Gain-of-function studies of KDM4B and PLK1 were performed to explore their involvements in OC development. In vivo experiments were conducted by inducing xenograft tumors in nude mice.

**Results:**

Poor expression of miR-545 was found in OC tissues and cells compared to the normal ones and it indicated unfavorable prognosis in patients. Overexpression of miR-545 suppressed growth, migration, invasion and angiogenesis of OC cells as well as the angiogenesis ability of HUVECs. miR-545 was found to target mRNAs of KDM4B and PLK1, while KDM4B promoted the transcription of the PLK1 promoter through demethylation of H3K9me3. Either overexpression of KDM4B or PLK1 partially blocked the inhibitory effects of miR-545 mimic on OC cell growth, especially the former one. The in vitro results were reproduced in vivo.

**Conclusion:**

This study evidenced that miR-545 suppresses progression of OC through mediating PLK1 expression by a direct binding and an indirect regulation involving KDM4B-mediated demethylation.

**Supplementary Information:**

The online version contains supplementary material available at 10.1186/s12885-021-07830-8.

## Background

Ovarian cancer (OC) is a frequent gynecological malignancy which accounts for 3.4% incidence and 4.4% mortality among all cancers in female with approximately 295,414 new cases and 184,799 deaths identified worldwide in 2018 [1]. Most OCs are originated in epithelium and therapeutic strategy prioritizes surgical resection and cytoreduction followed by cytotoxic chemotherapy such as platinum and taxane [2]. Early diagnosis is a decisive factor for better survival, since patients at advanced stages having an initial response to the conventional treatment are likely to develop recurrence during the clinical course [2, 3]. Unfortunately, due to the lack of specific symptoms, approximately 70% of patients are diagnosed at late stages and have a less than 50% overall 5-year survival rate [4]. Substantial efforts have been made by researchers in this field to develop new diagnostic and therapeutic tools or targets for OC control, while insightful understandings in the molecular mechanisms implicated in the disease progression are still needed.

MicroRNAs (miRNAs) have been increasingly accepted to impact cancer disease progression. They are small non-coding RNA molecules (approximately 22 nucleotides in length) abundantly existed in animals and plants with the primary function in mRNA silencing through regulation of gene expression post-transcriptionally [5]. miRNAs are closely linked to nearly every carcinogenesis process from tumor development to drug resistance in human cancers including OC [6]. Among the miRNAs, miR-545 has been reported with a tumor-suppressing role in another gynecological malignancy, cervical cancer, by suppressing proliferation and aggressiveness of cancer cells [7]. Likewise, miR-545 has been reported as a potential tumor suppressor that was poorly expressed in OC tissues [8]. These findings attracted our attention to validate the role of miR-545 in OC progression and the molecules involved. Polo-like kinase 1 (PLK1) and Lysine (K)-specific demethylase 4B (KDM4B), both of which have been reported to be highly expressed in OC cells and trigger cancer development [9, 10], were found as mRNA targets of miR-545 according to the data on a bioinformatic system StarBase (http://starbase.sysu.edu.cn/). KDM4B is a member of the KDM4/JMJD2 family of histone demethylases that regulate the catalytic activity against the histone residues with a preference to the H3K9me2/3 substrates which are related to gene repression [11]. Interestingly, KDM4B was found to activate the transcription of PLK1 through the demethylation modification of H3K9me3, therefore promoting the growth of prostate tumor [12]. This triggered us to explore if there is a similar regulatory work in OC. Hence, this study was performed to validate the potential interactions among miR-545, KDM4B and PLK1 and their functions on OC development by altering their expression in both cellular and animal experiments.

## Methods

### Clinical sample collection

Tumor and the adjacent normal ovarian tissues (over 1.5 cm away from the lesion sites) were obtained from 60 OC patients who were admitted into and underwent surgery in The Second Affiliated Hospital of Zhengzhou University from April 2012 to January 2014. All recruited patients were free of a history of radio- or chemo-therapy, and free of any other malignancies neither. The tissue samples were collected during surgery and then instantly preserved at − 80 °C. This study was conducted as per the *Declaration of Helsinki* and ratified by the Ethics Committee of The Second Affiliated Hospital of Zhengzhou University. Each eligible patient signed an informed consent.

### Cell preparation

OC cell lines (Caov3, OV-90, OVCAR3 and ES-2) acquired from ATCC (Manassas, VA, USA) were cultured in Dulbecco’s modified Eagle’s medium (DMEM, Invitrogen, Thermo Fisher Scientific, Rockford, IL, USA). A human immortalized ovarian epithelial cell line SV40 acquired from Applied Biological Materials Inc. (Richmond, Canada) was cultivated in Prigrow I medium (Abm, Richmond, Canada). All media were supplemented with 10% fetal bovine serum (FBS, Hyclone, Logan, UT, USA) and 1% penicillin/streptomycin (Gibco, Thermo Fisher) at 37 °C in air enriched by 5% CO_2_.

The miR-545 mimic, overexpressing vector of KDM4B (oe-KDM4B), oe-PLK1 and the corresponding negative controls (NC) used for transfection were all purchased from GenePharma Co., Ltd. (Shanghai, China). All transfections were conducted using a Lipofectamine 2000 kit (Invitrogen) in line with the manufacturer’s instructions. The transfection efficacy was determined 48 h later by reverse transcription quantitative polymerase chain reaction (RT-qPCR) or western blot assays.

### RT-qPCR

The TRIzol Reagent (Invitrogen) was used for extract total RNA from tissues and cells. RNA was reversely transcribed into cDNA using a miScript II RT kit (TaKaRa, Biotechnology Ltd., Dalian, China) and then the real-time qPCR was performed using a Premix Ex TaqTM II (TaKaRa) on an ABI PRISM 7500 real-time PCR System. Relative gene expression was determined by the 2^-ΔΔCt^ method. The primers are listed in Table 1, in which U6 and GAPDH were set as the internal references for miRNA and mRNAs, respectively.

**Table 1:** Primer sequences for RT-qPCR

Gene	Primer sequence (5′-3′)
miR-545	F: CGACAAGGGTCAGCAAACATT
	R: GCAGGGTCCGAGGTATTC
Bax	F: TCAGGATGCGTCCACCAAGAAG
	R: TGTGTCCACGGCGGCAATCATC
Bcl-2	F: ATCGCCCTGTGGAGAACTACTGAGT
	R: GCCAGGAGAAATCAAACAGAGGC
KDM4B	F: GCCGAGAGGAAGTTCAACGCAG
	R: TGCCTCCTTCTCAGAGTGTGTAGG
PLK1	F: GCACAGTGTCAATGCCTCCAAG
	R: GCCGTACTTGTCCGAATAGTCC
U6	F: CTCGCTTCGGCAGCACA
	R: AACGCTTCACGCATTTGC
GAPDH	F: GTCTCCTCTGACTTCAACAGCG
	R: ACCACCCTGTTGCTGTAGCCAA

Note: *RT-qPCR* reverse transcription quantitative polymerase chain reaction, *Bcl-2* B-cell lymphoma-2, *Bax* Bcl-2-associated X, *KDM4B* Lysine (K)-specific demethylase 4B, *PLK1* Polo-like kinase 1, *GAPDH* glyceraldehyde-3-phosphate dehydrogenase, *F* forward, *R* reverse

### 3-(4, 5-dimethylthiazol-2-yl)-2, 5-diphenyltetrazolium bromide (MTT) assay

Viability of cells was evaluated by the MTT assay. In brief, the transfected cells were sorted in 96-well plates at 3000 cells per well. Forty-eight hours later, each well was loaded with 20 μL MTT solution (5 mg/mL, Solarbio Science & Technology Co., Ltd., Beijing, China). Following another 3 h of incubation at 37 °C, the supernatant was discarded and the precipitated formazan was dissolved in 150 μL dimethyl sulphoxide (DMSO) solution. Then, the optical density (OD) value was determined at 490 nm using a microplate spectrophotometer.

### 5-ethynyl-2′-deoxyuridine (EdU) labeling assay

A Cell-Light EdU DNA replication kit (RiboBio Co., Ltd., Guangzhou, Guangdong, China) was used to evaluate cell proliferation. In brief, cells were sorted in 96-well plates (2 × 10^4^ cells/well). After 36 h, each well was filled with 50 μM EdU solution for another 3 h of incubation. Next, the cells were immobilized in formaldehyde and stained with Apollo solution. Hoechst 33342 was utilized to counterstain the nuclei. Images were obtained using an inverted fluorescence microscope (Olympus Optical Co., Ltd., Tokyo, Japan). The EdU-positive cells in 5 random fields were counted.

### Colony formation assay

Briefly, cells were sorted in 6-well plates at 500 cells per well for 2 weeks (w). Then the cells were fixed, stained with 1% crystal violet, and counted under the microscope.

### Terminal deoxynucleotidyl transferase (TdT)-mediated dUTP nick end labeling (TUNEL)

A TUNEL kit (Beyotime Biotechnology Co., Ltd., Shanghai, China) was used to measure cell apoptosis. The cells were fixed for 30 min (min) and then incubated in phosphate-buffered saline (PBS) containing 0.3% Triton X-100 for 5 min. After that, the cells were further treated with fresh TUNEL Reagent for 60 min of incubation at 37 °C without light exposure. Next, 4′, 6-diamidino-2-phenylindole (DAPI) was used for nuclear staining. Then, the cell slides were sealed by fluorescent mounting media and the relative fluorescence intensity was determined using an EVOS FL microscope (Invitrogen). The cell apoptosis rate was calculated as follows: apoptosis rate = TUNEL-positive cells (green fluorescence)/DAPI-labeled cells (blue fluorescence) × 100%.

### Western blot analysis

OC cells were lysed in cell lysis buffer (Beyotime) containing protease and phosphatase inhibitors to extract the total protein. The protein concentration was evaluated by a bicinchoninic acid (BCA) kit (Thermo Fisher). Then, 30 μg protein sample was run on 12% SDS-PAGE and transferred onto PVDF membranes (Millipore, Billerica, MA, USA). After being blocked in 5% bovine serum albumin, the membranes were cultured with the primary antibodies against E-cadherin (1:50, ab1416, Abcam, Inc., Cambridge, MA, USA), N-cadherin (1:5000, ab76011, Abcam), Vimentin (1:1000, ab92547, Abcam); PLK1 (1:500, #4535, Cell Signaling Technology (CST), Beverly, MA, USA), KDM4B (1:5000, ab191434, Abcam), H3K9me3 (1:1000, ab176916, Abcam), and GAPDH (1:5000, ab8245, Abcam) at 4 °C overnight. Then, the membranes were stained with the secondary antibodies anti-mouse IgG H&L (HRP) (1:10,000, ab205719, Abcam) and goat anti-rabbit IgG H&L (HRP) (1:10,000, ab205718, Abcam) at 37 °C for 45 min. The protein blots were developed by enhanced chemiluminescence (Millipore) and examined on a gel imaging system (Bio-Rad, Hercules, CA, USA). GAPDH was set as the control, and the signal intensity was examined by Image J.

### Scratch test

Transfected cells were sorted in 96-well plates with serum-free medium at 5 × 10^4^ cells per well and cultivated overnight. A scratch was produced on the cells using pipette tips. The scratch width was evaluated on the 0 h and 24th h under the inverted microscope.

### Transwell assay

A Transwell kit (Corning Incorporated, Corning, NY) was used for invasion detection. Each apical chamber pre-coated with Matrigel was loaded with 5 × 10^4^ cells in serum-free medium, while each basolateral chamber was loaded with 500 μL complete medium. The chambers were incubated at 37 °C for 24 h, and then the cells on the upper membrane were discarded, while the invaded cells were fixed and stained by 0.1% crystal violet. Then, the staining was observed under the microscope with 5 fields included.

### Angiogenesis assay

A total of 1.5 × 10^4^ human umbilical vein endothelial cells (HUVECs, ATCC) were sorted in Matrigel-coated 24-well plates and cultured with conditioned medium (CM) of different OC cells for 6 h. Then, the cells were observed and captured under the inverted microscope, and the number of formed tubes in 5 random fields was calculated and determined using the Image J software.

### Immunohistochemical (IHC) staining

Tumor tissues were fixed, embedded in paraffin, cut into 4-μm sections, dewaxed, and rehydrated. Then, the sections were soaked in 10 mmol/L prewarmed sodium citrate buffer (pH = 6.0) for 3 min of antigen retrieval, and then treated with 3% H_2_O_2_ for 10 min. Then, the sections were co-cultured with the primary antibodies against KDM4B (1:150, ab191434, Abcam), PLK1 (1:50, ab17056, Abcam), vascular endothelial growth factor A (VEGFA, 1:20, ab1316, Abcam) and Ki67 (1:20, ab21700, Abcam) at 4 °C overnight, and then with the goat anti-rabbit IgG H&L (HRP) (ab97051) or goat anti-mouse IgG H&L (HRP) (ab205719) at 37 °C for 45 min. Then, 3,3′-diaminobenzidine and hematoxylin reagent were used for color development. Then, the stained slides were imaged under a microscope, and the positive cells from 5 random fields were analyzed by the Image J. For the tissue samples from OC patients, the staining intensity was scored by three group-blinded pathologists according to a previous report [13]. The percentage of positive cells was allocated into five grades (positive scores): 0–3% (0), 3–25% (1), 26–50% (2), 51–75% (3) and 76–100% (4), and the staining density was divided into four grades (intensity scores): negative (0), weak brown (1), median brown (2) and strong brown (3). The final score of IHC staining was the product of two scores, which was allocated into three grades: negative (≤ 3), weak positive (3 < score ≤ 6) and strong positive (> 6).

### Dual-luciferase reporter gene assay

The wild type (WT) sequences of KDM4B mRNA (KDM4B-WT) and PLK1 mRNA (PLK1-WT) containing the putative binding site with miR-545, and the corresponding mutant type (MT) sequences (KDM4B-MT and PLK1-MT) based on the mutant binding sites were inserted into pmirGLO luciferase vectors (GeneCreate Biological Engineering Co., Ltd., Hubei, China). Then, 1 × 10^5^ 293 T cells (ATCC) were sorted in 24-well plates, and the well-constructed MT and WT vectors were co-transfected with either miR-545 mimic or NC mimic into the cells. Forty-eight hours later, the relative luciferase activity in cells was evaluated on a luciferase reporter gene system (Promega, Corp., Madison, Wisconsin, USA).

### Chromatin immunoprecipitation (ChIP)

All procedures were carried out according to the instructions of a ChIP kit (Millipore). In short, 1 × 10^7^ cells were crosslinked in 1% formaldehyde solution for 20 min. Then, the DNA fragments (200–500 bp) were obtained following ultrasonication. The lysates of OV-90 cells and ES-2 cells were precipitated by anti-KDM4B or anti-H3K9me2 with anti-IgG as control. The enrichment of PLK1 promoter in the immunoprecipitates was determined by RT-qPCR.

### Tumor growth and metastasis in mice

A total of 40 BALB/c nude mice (3–5 weeks old) from Vital River Laboratory Animal Technology Co., Ltd. (Beijing, China) were used for in vivo experiments. The animal experimental protocol was approved by the Ethics Committee of Animal Experiments of The Second Affiliated Hospital of Zhengzhou University. All animal procedures were performed in line with the Guide for the Care and Use of Laboratory Animals published by the National Institutes of Health (NIH, Bethesda, Maryland, USA). Great attempts were made to reduce the pain in animals. All animals were housed in a specific pathogen-free grade condition in a 12-h dark/light cycle at constant 22 ± 2 °C with 40% humidity. The mice were allowed to free access to water and feed. The complete formula feed sterilized via Co60 exposure and the drinking water were autoclaved. The feed and water were refreshed routinely twice a week and supplemented when necessary. The padding material was refreshed once a week. All procedures were performed by professional operators in sterile conditions.

For the tumor growth assay, 20 mice were assigned into 4 groups, and cells (1 × 10^5^/mL) with stable transfection of miR-545 mimic or NC mimic were implanted into the ventral side of mice through subcutaneous injection. Ten days (d) after transfection, the volume (V) of xenograft tumors was evaluated every 5 d using the following formula: V = L × W^2^/2, where L indicates the length while W indicates the width. After 35 d, the mice were euthanized through an intraperitoneal injection of pentobarbital sodium (150 mg/kg), and the tumors were collected for weight and volume measurement. The tumor tissues were further used for IHC staining. The rest 20 mice were used for metastasis assay. In brief, cells with stable transfection (1 × 10^7^/mL) were administrated into the mice through the caudal veins. The mice were euthanized on the 45th d, and then the lung tissues were collected for hematoxylin and eosin (HE) staining. The animal carcasses were collected as medical wastes and burned.

### HE staining

The lung tissues of the nude mice were fixed in formaldehyde, dehydrated, embedded, and cut into sections. Then, the sections were dewaxed in xylene, rehydrated, and then stained with hematoxylin for 4 min and then with eosin for 1 min. After that, the sections were dehydrated, cleared, sealed, and observed under the microscope. The relative metastatic area in lung tissues was calculated using Image J.

### Statistical analysis

Data were analyzed using Prism 8.0 (GraphPad, La Jolla, CA, USA). Data were presented as mean ± standard deviation (SD) from no less than three independent experiments. Differences were analyzed by *t* test (two groups) and one-way or two-way analysis of variance (ANOVA), followed by Tukey’s multiple test (over 2 groups). The Log-rank (Mantel-Cox) test was used to analyze the 5-year survival rate of patients. The correlations between variables were evaluated by Pearson’s correlation analysis. Clinical characteristics of patients were analyzed using Fisher’s exact test. *p < 0.05 was regarded to present statistical significance.

## Results

### miR-545, positively linked to the survival of patients, is poorly expressed in OC tissues and cells

**Fig. 1:**
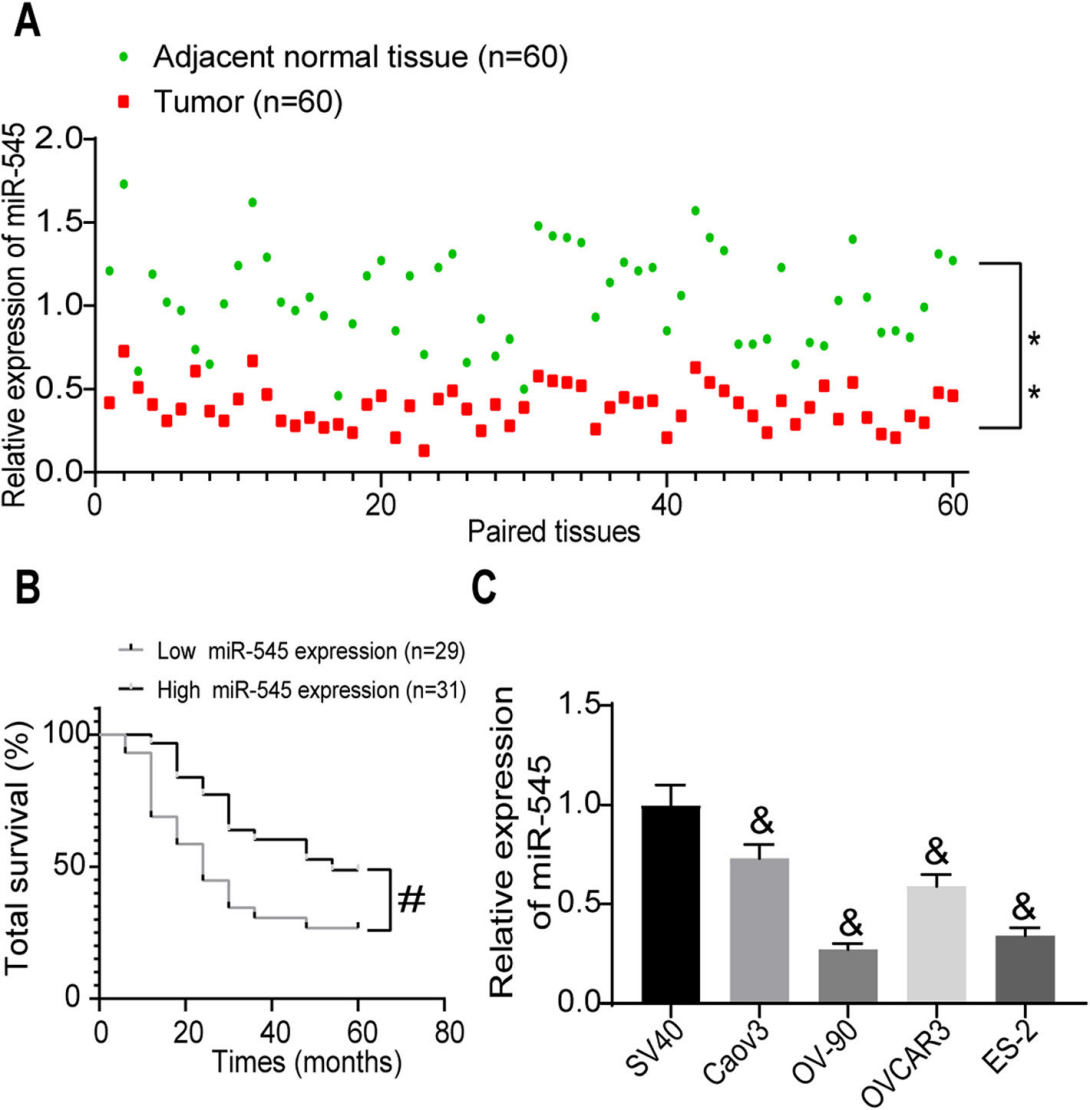
miR-545 is poorly expressed in OC tissues and cells. **a** expression of miR-545 in OC and the adjacent tissues determined by RT-qPCR (*n* = 60, paired *t* test, **p < 0.01); **b** correlation between miR-545 level and the 5-year survival rate of OC patients (Log-rank Mantel-Cox) test, #*p* < 0.05); **c** miR-545 expression in OC cell lines (Caov3, OV-90, OVCAR3 and ES-2) and in SV40 cells determined by RT-qPCR (one-way ANOVA, &*p* < 0.05). Data were collected from three independent experiments and expressed as mean ± SD

We first determined the expression of miR-545 in the collected tumor and normal tissues from the 60 patients. The RT-qPCR results suggested that miR-545 was poorly expressed in the tumor samples relative to the paired adjacent normal tissues (Fig. 1a). Then, the patients were allocated into high miR-545 expression group (*n* = 31) and low miR-545 expression group (*n* = 29) according to the average value of miR-545 (0.39). Then, the follow-up study results showed that patients with high miR-545 expression had a notable higher survival rate than those with low miR-545 expression (Fig. 1b). The correlation between miR-545 expression and the clinical characteristics of patients was analyzed. Poor expression of miR-545 was found to be relevant to advanced pathologic tumor-node metastasis (pTNM) staging (Supplementary Table 1). Additionally, expression of miR-545 in the OC cell lines and in SV40 cells was measured. Likewise, poor expression of miR-545 was found in the Caov3, OV-90, OVCAR3 and ES-2 cells compared to the SV40 cells (Fig. 1c). Among them, OV-90 and ES-2 cells having lowest levels of miR-545, were selected for the subsequent experiments.

### Upregulation of miR-545 suppresses growth of OC cells

**Fig. 2:**
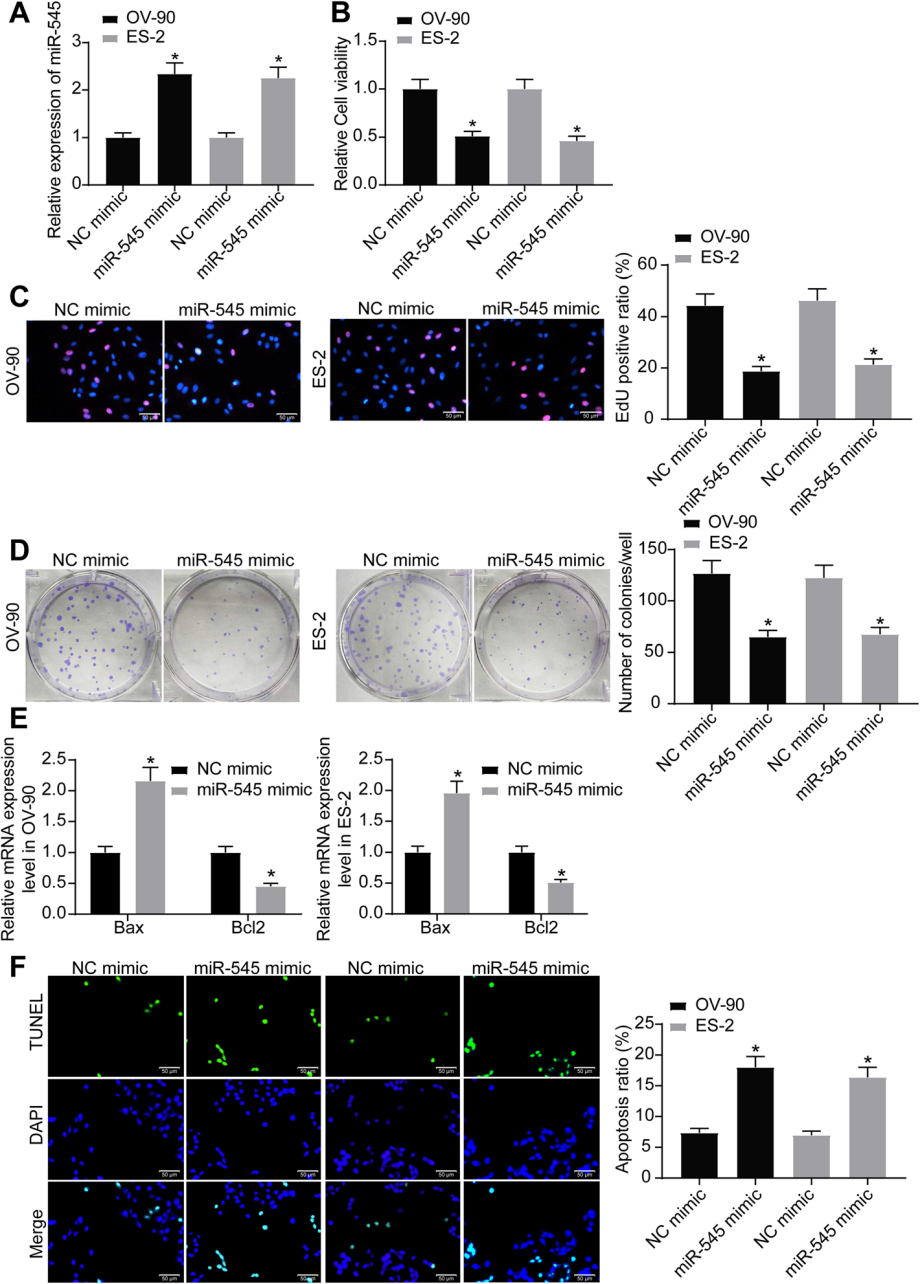
Upregulation of miR-545 suppresses growth of OC cells. **a** miR-545 expression in cells following miR-545 mimic transfection determined by RT-qPCR (one-way ANOVA, *p < 0.05); **b** viability of cells determined by the MTT assay (one-way ANOVA, *p < 0.05); **c** DNA replication ability of cells evaluated by the EdU assay (one-way ANOVA, *p < 0.05); **d** proliferation of cells measured by the colony formation assay (one-way ANOVA, *p < 0.05); **e** mRNA expression of Bax and Bcl-2 in cells determined by RT-qPCR (one-way ANOVA, *p < 0.05); **f** apoptosis of cells determined by TUNEL assay (one-way ANOVA, *p < 0.05). Representative images are provided. Data were collected from three independent experiments and expressed as mean ± SD

To further explore the function of miR-545 on OC development, in vitro experiments were first performed where miR-545 mimic was transfected into OV-90 and ES-2 cells, after which the miR-545 expression in cells was successfully increased (Fig. 2a). Thereafter, the MTT assay results showed that the viability of cells was notably suppressed by miR-545 mimic (Fig. 2b). Likewise, the EdU assay suggested that the DNA replication ability, namely proliferation of cells, was reduced by miR-545 mimic (Fig. 2c). More obviously, the number of colonies of OC cells, according to the colony formation assay, was significantly inhibited upon miR-545 upregulation as well (Fig. 2d).

In terms of cell apoptosis, we first determined the expression of apoptosis-related cytokines Bax and Bcl-2 in cells. It was found that miR-545 led to an increase in the expression of pro-apoptotic Bax while a decline in the anti-apoptotic Bcl-2 (Fig. 2e). In addition, the TUNEL results showed that miR-545 mimic significantly promoted the apoptosis of OV-90 and ES-2 cells (Fig. 2f).

### Upregulation of miR-545 blocks migration, invasion and angiogenesis of OC cells

**Fig. 3:**
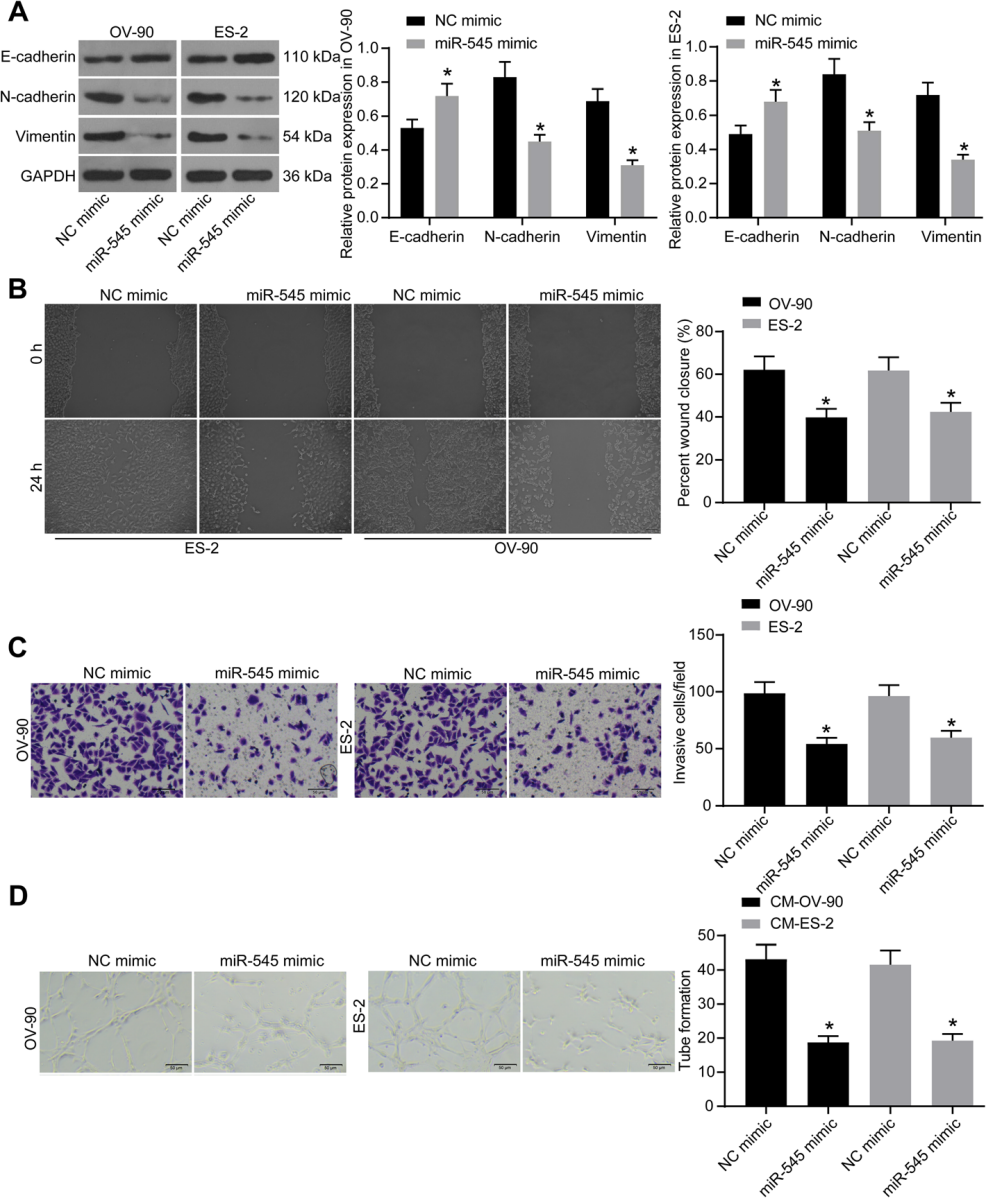
Upregulation of miR-545 blocks migration, invasion, and angiogenesis of OC cells. **a** relative expression of the EMT-related proteins E-cadherin, N-cadherin and Vimentin relative to GAPDH measured by western blot analysis (two-way ANOVA, *p < 0.05); **b**-**c** migration (**b**) and invasion (**c**) abilities of cells determined by scratch test and Transwell assay, respectively; **d** angiogenesis ability of HUVECs determined by tube formation assay (one-way ANOVA, *p < 0.05). Repetition = 3. Representative images are provided. Data were collected from three independent experiments and expressed as mean ± SD

The role of miR-545 in the metastatic potential of OC cells was further determined. First, western blot analysis was applied to explore the expression of epithelial to mesenchymal cell transition (EMT)-related marker proteins E-cadherin, N-cadherin and Vimentin in cells. The results showed that miR-545 increased the level of the epithelial marker E-cadherin while decreased the levels of mesenchymal markers N-cadherin and Vimentin (Fig. 3a), indicating that miR-545 has the potential to suppress EMT activity of OC cells.

Next, the direct links between miR-545 and the migration as well as invasion of the OV-90 and ES-2 cells were investigated. The scratch test suggested that the wound-healing, namely the migration ability of cells was suppressed upon miR-545 upregulation (Fig. 3b). Similarly, the Transwell assay results showed that the invasive potential of cells was suppressed by miR-545 mimic as well (Fig. 3c). Then, an angiogenesis assay was performed, in which HUVECs were cultured in the CM of OC cells transfected with miR-545 mimic or NC mimic. Consequently, it was found that miR-545 mimic suppressed the angiogenesis ability of HUVECs (Fig. 3d).

### miR-545 directly binds to PLK1 mRNA

**Fig. 4:**
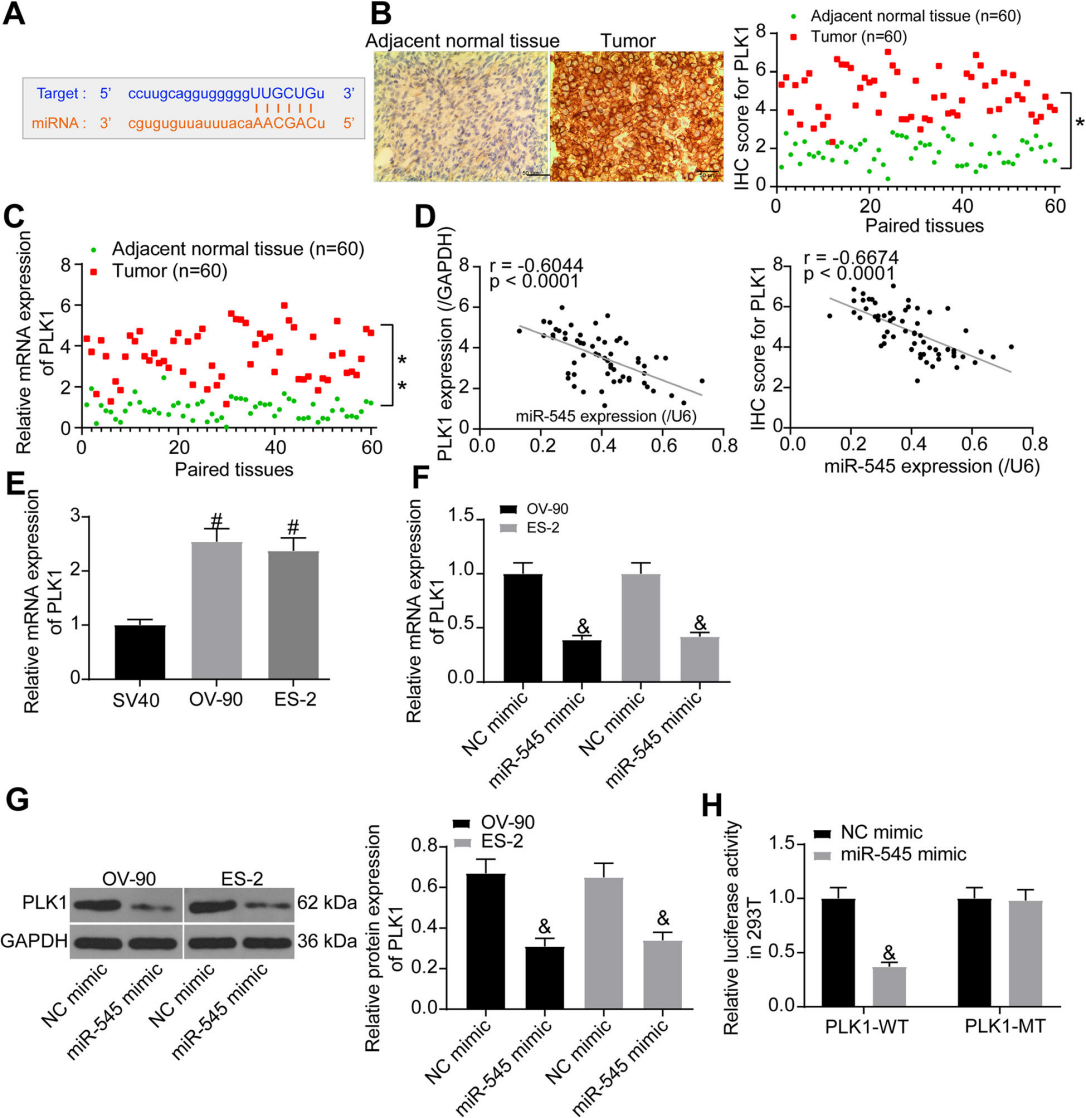
miR-545 directly binds to PLK1 mRNA. **a** putative binding site between miR-545 and PLK1 predicted on Starbase; **b** PLK1 expression in OC and the paired normal tissues determined by IHC staining (*n* = 60, paired *t* test, *p < 0.05); **c** mRNA expression of PLK1 in the tissues determined by RT-qPCR (*n* = 60, paired *t* test, **p < 0.01); **d** a negative correlation between miR-545 and PLK1 expression (Pearson’s correlation analysis, miR-545 vs. PLK1 mRNA: *r* = − 0.6044, *p* < 0.0001; miR-545 VS IHC staining score of PLK1: *r* = − 0.6674, *p* < 0.0001); **e** PLK1 expression in SV40, OV-90 and ES-2 cells measured by RT-qPCR (one-way ANOVA, #*p* < 0.05); **f**-**g** mRNA (**f**) and protein (**g**) expression of PLK1 in OV-90 and ES-2 cells after miR-545 mimic administration measured by RT-qPCR and western blot analysis, respectively (relative to GAPDH, one-way ANOVA, &*p* < 0.05); **h** binding relationship between miR-545 and PLK1 validated by a dual luciferase reporter gene assay (two-way ANOVA, &*p* < 0.05). Representative images are provided. Data were collected from three independent experiments and expressed as mean ± SD

To further explore the downstream molecules involved, we first predicted the targeting mRNAs of miR-545. PLK1, which has been noted to trigger the growth and aggressiveness of OC cells [9], was found as a target of miR-545 (Fig. 4a). Thereafter, the IHC staining results suggested that the expression of PLK1 in OC tissues was notably higher than that in the adjacent tissues (Fig. 4b). Likewise, the RT-qPCR results presented a similar trend that the mRNA expression of PLK1 was increased in OC tissues as well (Fig. 4c). Either mRNA or protein expression of PLK1 showed a negative correlation with the miR-545 expression (Fig. 4d). Still, high expression of PLK1 was also identified in the OV-90 and ES-2 cells compared to the SV40 cells (Fig. 4e). Further, we noticed that high expression of miR-545 in cells led to a notably decline in PLK1 mRNA expression (Fig. 4f). Similarly, the protein level of PLK1 in these two cell lines, was suppressed by miR-545 mimic (Fig. 4g). In addition, a luciferase reporter assay was performed to validate the direct link between miR-545 and PLK1. It was found that co-expression of miR-545 mimic and PLK1-MT vector led to a notable decline in the luciferase activity in 293 T cells, while transfection of NC mimic or PLK1-MT vector had no effects (Fig. 4h).

### miR-545 directly targets KDM4B mRNA

**Fig. 5:**
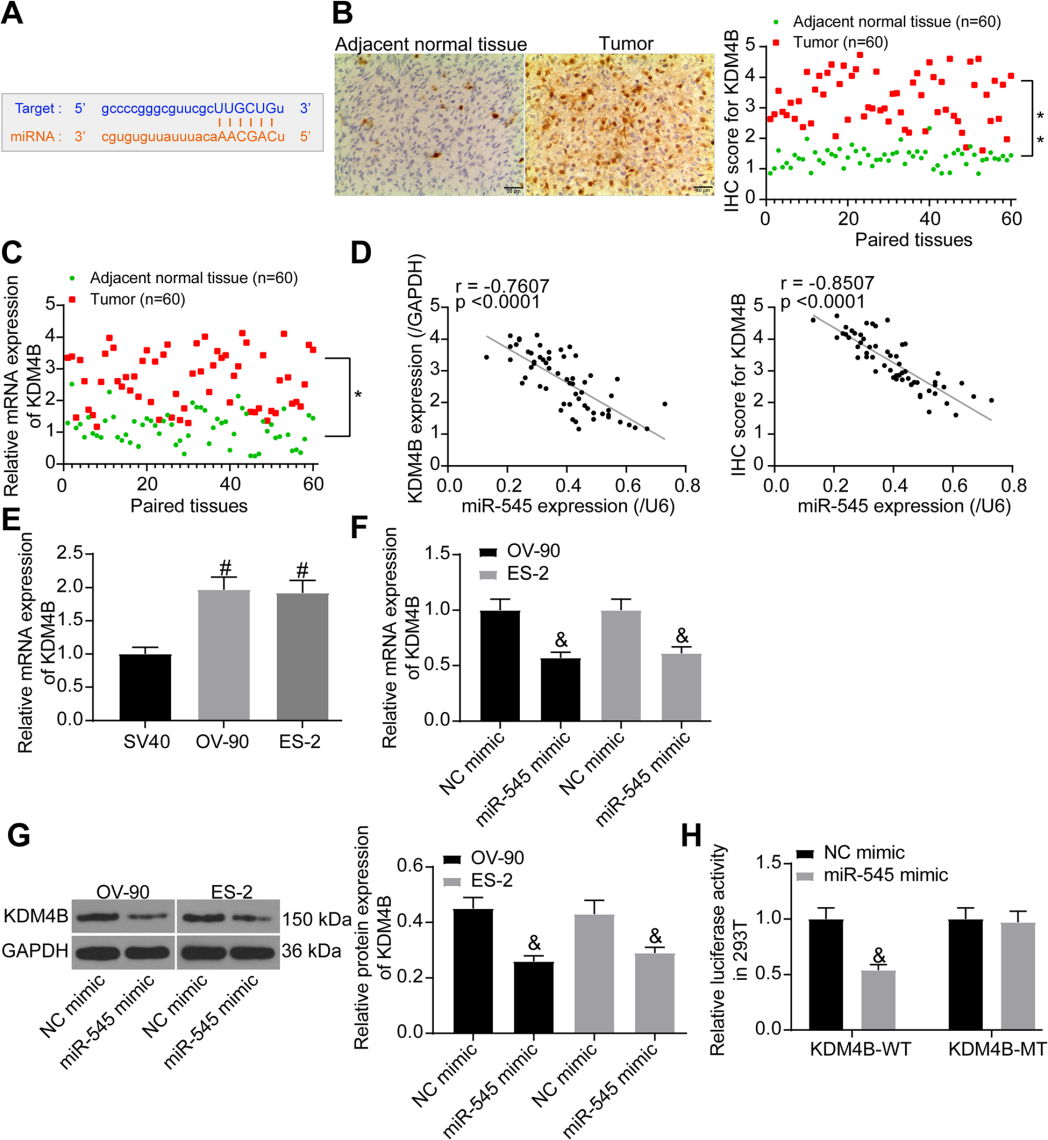
miR-545 directly targets KDM4B mRNA. **a** putative binding site between miR-545 and KDM4B predicted on Starbase; **b** KDM4B expression in OC and the paired normal tissues determined by IHC staining (*n* = 60, paired *t* test, *p < 0.05); **c** mRNA expression of KDM4B in the tissues determined by RT-qPCR (*n* = 60, paired *t* test, **p < 0.01); **d** a negative correlation between miR-545 and KDM4B expression (Pearson’s correlation analysis, miR-545 vs. KDM4B mRNA: *r* = − 7607, *p* < 0.0001; miR-545 VS IHC staining score of KDM4B: *r* = − 0.8507, *p* < 0.0001); **e** KDM4B expression in SV40, OV-90 and ES-2 cells measured by RT-qPCR (one-way ANOVA, #*p* < 0.05); **f**-**g** mRNA (**f**) and protein (**g**) expression of KDM4B in OV-90 and ES-2 cells after miR-545 mimic administration measured by RT-qPCR and western blot analysis, respectively (relative to GAPDH, one-way ANOVA, &*p* < 0.05); **h** binding relationship between miR-545 and KDM4B validated by a dual luciferase reporter gene assay (two-way ANOVA, &*p* < 0.05). Representative images are provided. Data were collected from three independent experiments and expressed as mean ± SD

Intriguingly, KDM4B, which has been reported as a highly expressed demethylase in OC [10] and a positive regulator of PLK1 [12], was identified as a putative target of mR-545 as well (Fig. 5a). Therefore, we speculated whether miR-545 also mediates PLK1 expression through an indirect way with the mediation by KDM4B.

Again, IHC staining was first performed and identified significantly high expression of KDM4B in OC tissues relative to the paired normal ones (Fig. 5b). Likewise, the mRNA expression of KDM4B was increased in OC tissues (Fig. 5c). The KDM4B expression also presented a negative correlation with the miR-545 expression (Fig. 5d). High expression of KDM4B was also confirmed in OV-90 and ES-2 cells relative to the SV40 cells (Fig. 5e).

According to the RT-qPCR and western blot assays, the mRNA and protein expression of KDM4B in OC cells was significantly decreased by miR-545 mimic (Fig. 5f-g). The binding relationship between miR-545 and KDM4B mRNA was validated by a luciferase assay in a similar manner as aforementioned, which suggested that the luciferase activity in 293 T cells was notably decreased following co-transfection of miR-545 mimic and the KDM4B-WT vector (Fig. 5h).

### KDM4B modifies H3K9me3 demethylation to promote PLK1 expression

**Fig. 6:**
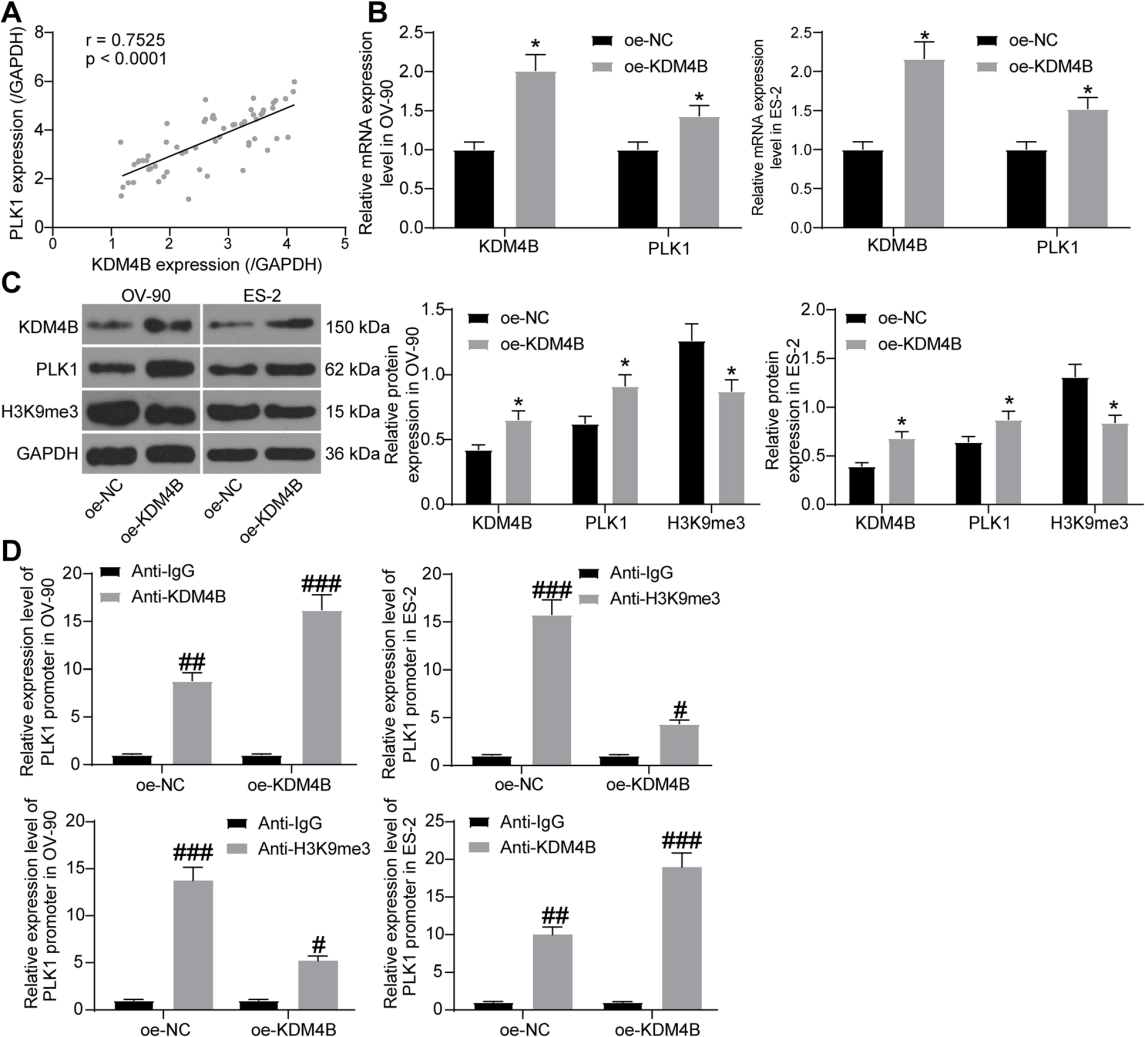
KDM4B modifies H3K9me3 demethylation to promote PLK1 expression. **a** correlation between KDM4B and PLK1 expression in OC tissues (Pearson’s correlation analysis, *r* = 0.7525, *p* < 0.0001); **b** mRNA expression of KDM4B and PLK1 in OC cells after oe-KDM4B administration determined (two-way ANOVA, *p < 0.05); **c** protein expression of KDM4B, PLK1 and H3M9me3 in OC cells measured by western blot analysis (relative to GAPDH, two-way ANOVA, *p < 0.05); **d** enrichment of the PLK1 promoter by anti-KDM4B or anti-H3K9me3 relative to anti-IgG determined by a ChIP assay (two-way ANOVA, #*p* < 0.05, ##*p* < 0.01, ###*p* < 0.001). Representative images are provided. Data were collected from three independent experiments and expressed as mean ± SD

The above results showed that miR-545 could target KDM4B and PLK1. Interestingly, the inhibitory effect of miR-545 on PLK1 seemed to be stronger than that on KDM4B (miR-545 mimic had a 2.5-fold inhibitory effect on PLK1 mRNA and a 2.3-fold effect on PLK1 protein compared to NC mimic; while that on the mRNA and protein of KDM4B were 1.7 and 1.6 folds, respectively). We speculated that this might be owing to the potential regulation of PLK1 by KDM4B. Thereafter, a positive correlation between KDM4B and PLK1 was identified in OC tissues (Fig. 6a). Then, oe-KDM4B was transfected into OC cells, after which the RT-qPCR and western blot assays confirmed that the expression of KDM4B was notably increased (Fig. 6b-c). In addition, the mRNA and protein expression of PLK1 was increased by oe-KDM4B as well, while the methylation of H3K9me3 was significantly reduced (Fig. 6b-c). Following these findings, a ChIP assay was further performed (Fig. 6d). Importantly, in OV-90 and ES-2 cells, an enrichment of PLK1 promoter sequence was found in the immunoprecipitates pulled down by anti-KDM4B or anti-H3K9me3 compared to anti-IgG, and the enrichment of PLK1 fragments by anti-KDM4B was further strengthened while the enrichment of PLK1 fragments by anti-H3K9me3 was reduced when cells were transfected with oe-KDM4B. Collectively, these results showed that KDM4B modifies demethylation of H3K9me3 and suppresses the inhibition of H3K9me3 on PLK1 promoter, therefore triggering the transcription of PLK1.

### Overexpression of KDM4B or PLK1 blocks the inhibitory effect of miR-545 on OC cell growth

**Fig. 7:**
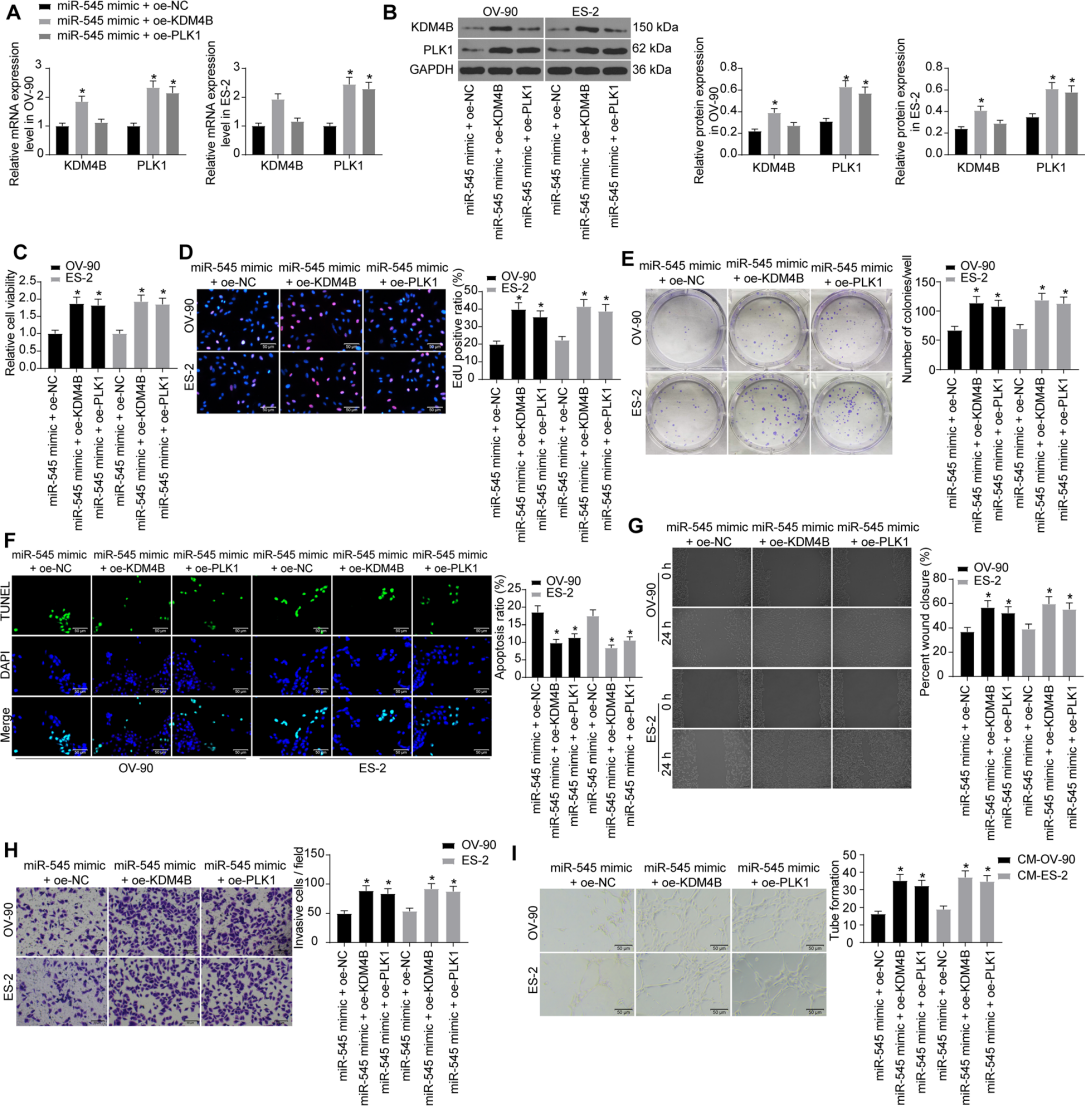
Overexpression of KDM4B or PLK1 blocks the inhibitory effect of miR-545 on OC cell growth. **a**-**b** mRNA (**a**) and protein (**b**) expression of KDM4B and PLK1 in OC cells transfected with miR-545 mimic and the additional oe-KDM4B or oe-PLK1 determined by RT-qPCR and western blot analysis, respectively (relative to GAPDH, two-way ANOVA, compared to miR-545 mimic + oe-NC, *p < 0.05); **c** viability of cells determined by MTT assay (two-way ANOVA, compared to miR-545 mimic + oe-NC, *p < 0.05); **d** DNA replication ability of cells measured by EdU labeling assay (two-way ANOVA, compared to miR-545 mimic + oe-NC, *p < 0.05); **e** proliferation of cells detected by colony formation assay (two-way ANOVA, compared to miR-545 mimic + oe-NC, *p < 0.05); **f** apoptosis rate of cells measured by TUNEL assay (two-way ANOVA, compared to miR-545 mimic + oe-NC, *p < 0.05); **g**-**h** migration (**g**) and invasion (**h**) abilities of cells determined by the scratch test and Transwell assay, respectively (two-way ANOVA, compared to miR-545 mimic + oe-NC, *p < 0.05); **i** angiogenesis ability of HUVECs determined by tube formation assay (two-way ANOVA, compared to miR-545 mimic + oe-NC, *p < 0.05). Representative images are provided. Data were collected from three independent experiments and expressed as mean ± SD

To further confirm the involvements of PLK1 and KDM4B in the events mediated by miR-545, oe-PLK1 or oe-KDM4B was additionally transfected in OC cells after miR-545 transfection. Then, according to the RT-qPCR and western blot assays, further administration of oe-KDM4B significantly elevated the expression of KDM4B and PLK1, and oe-PLK1 successfully increased PLK1 expression in cells (Fig. 7a-b). Interestingly, although the change was not significant, oe-PLK1 also moderately increased the expression KDM4B.

Then, the functions of oe-KDM4B and oe-PLK1 on OC cell growth were determined. The MTT and EdU labeling assays suggested that the viability and DNA replication ability of OC cells were significantly increased (Fig. 7c-d), and the colony formation ability of the cells was increased as well upon KDM4B or PLK1 overexpression (Fig. 7e). In addition, the increased cell apoptosis by miR-545 was further blocked by oe-KDM4B or oe-PLK1 according to the TUNEL assay (Fig. 7f).

The changes in metastatic potential in cells were also determined. The scratch test and Transwell assays suggested that both the migration (Fig. 7g) and invasion (Fig. 7h) abilities of cells suppressed by miR-545 mimic were partly recovered by oe-KDM4B or oe-PLK1. Besides, overexpression of KDM4B or PLK1 in OC cells led to an increase in the angiogenesis ability of HUVECs cultured in the corresponding CM of OC cells (Fig. 7i). Collectively, these results suggested that overexpression of KDM4B or PLK1 blocked the suppressive functions of miR-545 on OC cell growth and migration, and oe-KDM4B had a stronger effect.

### miR-545 mimic suppressed tumor growth and metastasis in nude mice

**Fig. 8:**
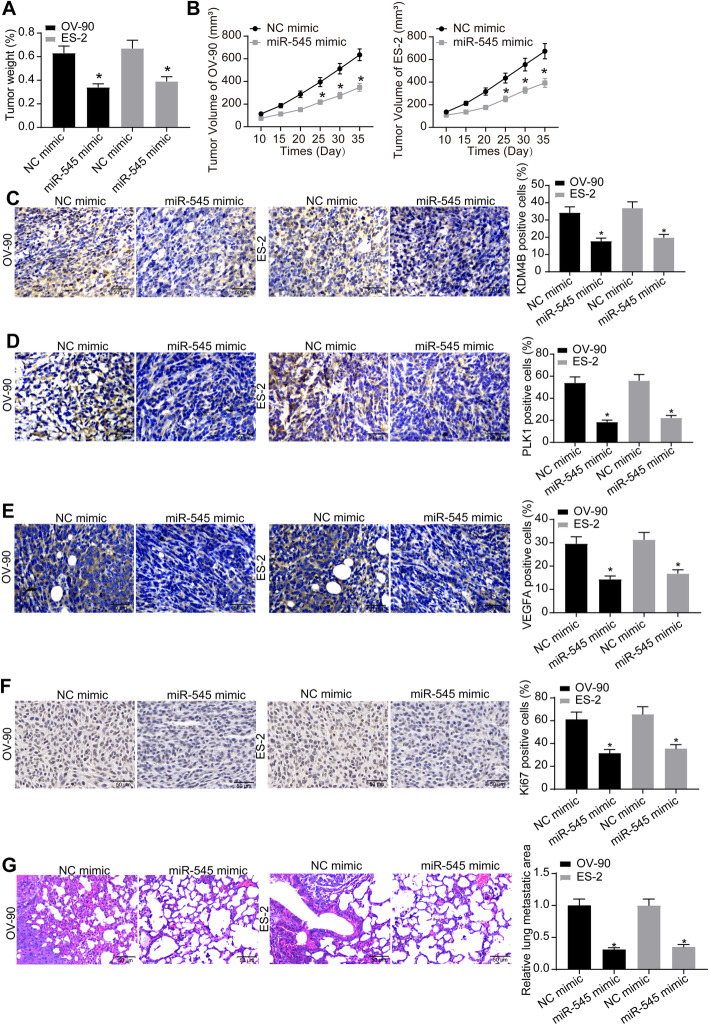
miR-545 mimic suppresses tumor growth and metastasis in nude mice. **a**-**b** weight (**a**) and volume (**b**) of the xenograft tumors in mice on the 35th d after cell implantation (*n* = 5 in each group, one-way ANOVA, *p < 0.05); **c**-**e** expression of KDM4B (**c**), PLK1 (**d**) and VEGFA (**e**) in cells determined by IHC staining (one-way ANOVA, *p < 0.05); **f** expression of Ki67 in the collected xenograft tumor tissues examined by IHC staining (one-way ANOVA, *p < 0.05); **g** area of the lung populated with cancer cells determined by HE staining (*n* = 5 in each group, one-way ANOVA, *p < 0.05). Representative images are provided. Data were collected from three independent experiments and expressed as mean ± SD

Following the findings above, the role of miR-545 in tumor growth in vivo was further investigated. OC cells with stable miR-545 mimic transfection were implanted into the mice through subcutaneous injection (for growth assay) or caudal vein injection (for metastasis assay). In terms of tumor growth, the mice were euthanized on the 35th d and the tumor tissues were collected. It was found that the weight and volume of the xenograft tumors were notably decreased upon miR-545 upregulation (Fig. 8a-b). The tissues were collected for IHC staining, which suggested that the positive rates of KDM4B and PLK1 as well as VEGFA were reduced by miR-545 mimic (Fig. 8c-e). In addition, miR-545 mimic also suppressed the expression of Ki67, a proliferation marker protein, in the xenograft tumor tissues (Fig. 8f). For the metastasis assay, the mice were euthanized on the 45th d, and the HE staining results suggested that the area of the lung populated with cancer cells was notably reduced (Fig. 8g).

## Discussion

Despite the surgical debulking and the administration of multiple antitumor treatment combinations, the overall 5-year survival rate of OC patients at advanced stages was no more than 40% with only modest improvement over decades [14]. In addition, the traditional approaches of treatment largely depend on individuals’ health condition and medical history, and are often linked to side-effects including hair loss, fatigue, neuropathies, nausea, and inflammatory bowel disease [15]. Developing less-invasive and effective options for OC control has been a crucial issue. In the current research, we identified a potent inhibitory function of miR-545 on OC cell and tumor growth through the suppression of PLK1 by direct binding or an indirect regulation by abrogating KDM4B-mediated PLK1 activation.

It has been well-established that altered expression of miRNAs is frequently associated with the ovarian tumorigenesis, leaving them as promising targets or markers for detection, prognosis and therapy [16]. Either anti-oncogenic [17–21] or oncogenic [22–24] roles of miRNAs have been recognized in OC. In this study, we first noticed that miR-545 was poorly expressed in the collected tumor tissues from OC patients and the acquired OC cell lines Caov3, OV-90, OVCAR3, and ES-2 compared to the normal ones. In addition, the follow-up study indicated that patients with higher miR-545 expression showed a longer survival time. These were in concert with the finding in a previous study by Jia et al., in which they found OC patients (sample volume = 27) expressing increased levels of miR-545 in epithelial OC tissues having increased survival rates [8]. In this study, overexpression of miR-545 suppressed viability and proliferation, migration and invasion of OC cells and angiogenesis of HUVECs, while promoted OC cell apoptosis of cells. In a molecular perspective, miR-545 overexpression led to an increase to the Bax/Bcl-2 ratio, as well as an increase in E-cadherin expression while a decline in the expression of N-cadherin and Vimentin. The tumor-suppressing role of miR-545 has been well demonstrated, such as suppressing cell proliferation and metastasis in cervical cancer [7], gastric cancer [25], colon adenocarcinoma [26] and so forth, and enhancing the radiosensitivity of Lewis xenograft tumor [27]. These reports suggested miR-545 as a promising and specific miRNA for cancer management. Here, combining with the results from animal experiments that upregulation of miR-545 suppressed the weight, volume, and lung metastasis of xenograft tumors, we reported a similar anti-tumor function of miR-545 in OC both in vitro and in vivo.

miRNAs exert their versatile functions primarily by binding to the mRNA targets to induce gene repression. In the present study, we noticed PLK1 and KDM4B as two important potential target genes of miR-545 according to the bioinformatics prediction, and the binding relationships were validated through luciferase reporter gene assays. PLK1 is a well-recognized oncogene that has been summarized in many key cellular processes from cell cycle progression, cell invasion and migration, and proliferation [28]. As aforementioned, PLK1 has been implicated in OC progression as well [9]. Likewise, activation of PLK1 was responsible for the oncogenic role of Aurora Borealis in OC [29]. PLK1 inhibitors have seen potentials in the clinical management of cancers by blocking cancer cell mitosis and triggering cell apoptosis [30]. In addition, although it has not been completedly translated to clinical trials, a specific PLK1 inhibitor, onvansertib, has shown good tolerance and efficacy in combination therapy with decitabine for leukemia treatment [31]. High expression of PLK1 has been reported to link to poor prognosis in patients, and targeting inhibition of PLK1 in combination with paclitaxel and proTAME has shown potent effects on the reduction of chromosomal instability and the subsequent apoptosis of OC cells [32]. These results indicated that miR-545-mediated PLK1 downregulation has potentials in OC control. In addition, epigenetic changes are well-recognized contributors to tumorigenesis and normal developmental processes [11]. KDM4B is a member of the KDM4/JMJD2 family that target H3K9me2/3 and H3K36me3 and are frequently overexpressed in human cancer cells and neoplastic tissues [33]. H3K9me3 is normally correlated with facultative heterochromatins, which are condensed and transcriptionally silent but can decondense upon H3K9me3 decline and become transcriptionally nonrestrictive in response to specific environmental and developmental cues [12]. Here, we observed that a similar KDM4B-H3K9me3-PLK1 regulatory work in OC cells through a ChIP assay, which was further evidenced by the expression examination where overexpression of KDM4B led to an increase in PLK1 expression in OC cells. Intriguingly, upregulation of PLK1 in OC cells elevated KDM4B expression in a moderate extend, though not significantly. This might be caused by the increased binding between miR-545 and PLK1 mRNA, and correspondingly, reduced binding with KDM4B. Then, we found that either overexpression of KDM4B or PLK1 blocked the inhibitory roles of miR-545 on OC cells, especially the former one. Similarly, in the animal models, miR-545 upregulation led to a decline in the expression of KDM4B and PLK1 in the xenograft tumors of mice.

## Conclusion

**Fig. 9:**
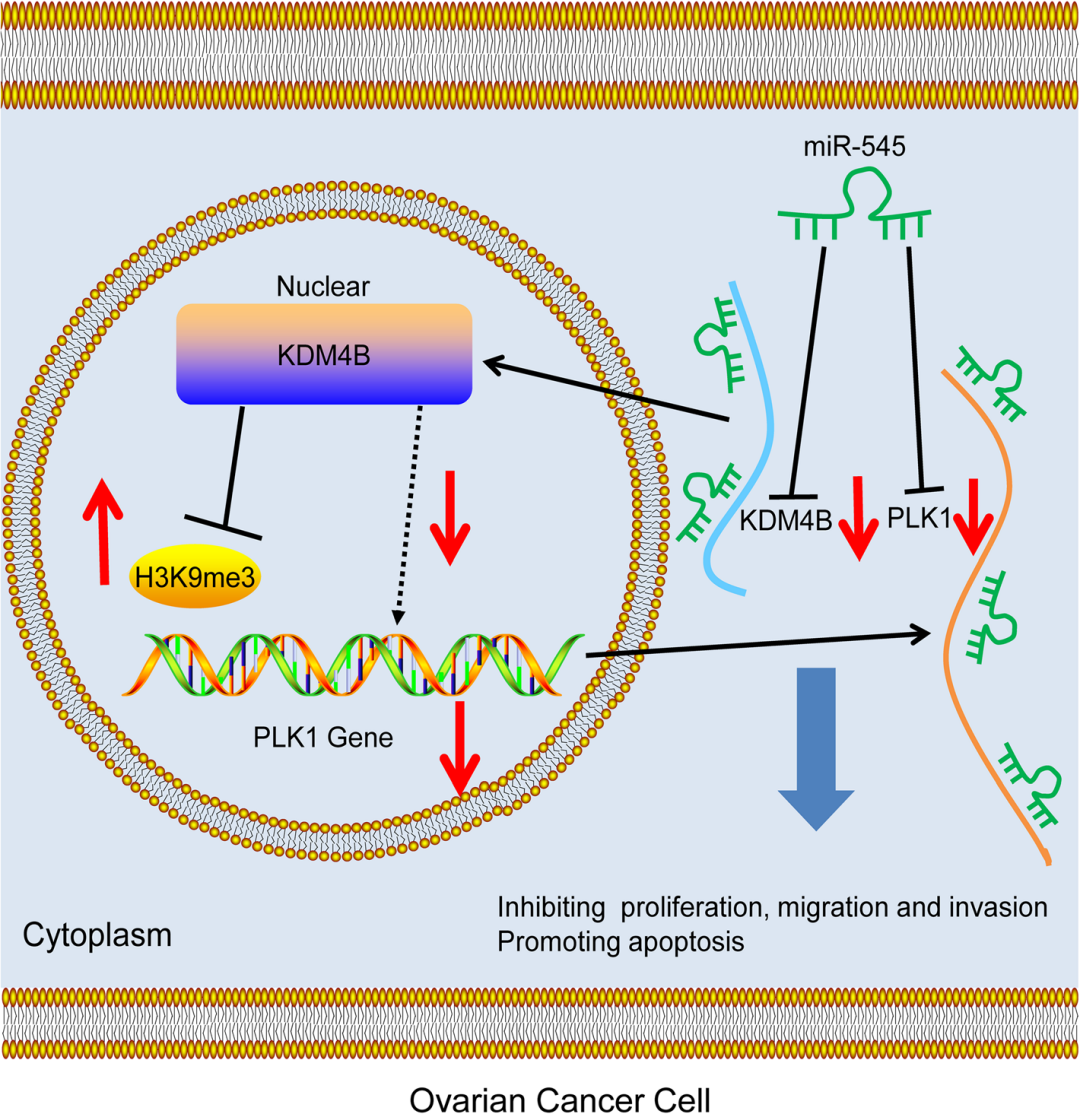
Diagram presentation of the molecular mechanism. KDM4B promotes the demethylation of H3K9me3 to reduce the transcriptional suppression of PLK1, while miR-545 directly binds to PLK1 and KDM4B, therefore suppressing PLK1 expression through a direct targeting manner and an indirect regulation through KDM4B, thus alleviating OC development

To sum up, this study evidenced that miR-545 exerts an anti-oncogenic role in OC through the direct suppression on PLK1 and KDM4B (Fig. 9). This study may offer novel insights into OC treatment. We would like to further investigate the potential pathways involved or activated following these events in our future studies. We also hope more researches in this field will be carried out to probe more ideas for OC control.

## Supplementary Information

**Additional file 1.**

## Data Availability

All the data generated or analyzed during this study are included in this published article.

## Abbreviations

ANOVA: Analysis of variance
ATCC: American Type Culture Collection
BCA: Bicinchoninic acid
ChIP: Chromatin immunoprecipitation
CM: Conditioned medium
DAPI: 4′, 6-diamidino-2-phenylindole
DMEM: Dulbecco’s modified Eagle’s medium
EdU: 5-ethynyl-2′-deoxyuridine
FBS: Fetal bovine serum
GAPDH: Glyceraldehyde-3-phosphate dehydrogenase
HE staining: Hematoxylin and eosin staining
HRP: Horseradish peroxidase
HUVECs: Human umbilical vein endothelial cells
IgG: Immunoglobulin G
KDM4B: Lysine (K)-specific demethylase 4B
mean ± SD: Mean ± standard deviation
miRNAs: MicroRNAs
MT: Mutant-type
MTT: 3-(4, 5-dimethylthiazol-2-yl)-2, 5-diphenyltetrazolium bromide
NC: Negative control
OC: Ovarian cancer
PBS: Phosphate buffer saline
PLK1: Polo-like kinase 1
PVDF: Polyvinylidene fluoride
RT-qPCR: Reverse transcription quantitative polymerase chain reaction
SDS-PAGE: Sodium dodecyl sulfate-polyacrylamide gel electrophoresis
TUNEL: Terminal deoxynucleotidyl transferase (TdT)-mediated dUTP nick end labeling
VEGFA: Vascular endothelial growth factor A
WT: Wild-type
