# Supplementary material for: MicroRNA-545 suppresses progression of ovarian cancer through mediating PLK1 expression by a direct binding and an indirect regulation involving KDM4B-mediated demethylation
Source: BMC Cancer. 2021 Feb 15;21:163. doi: 10.1186/s12885-021-07830-8 (PMC7885496; doi:10.1186/s12885-021-07830-8)
Supplement: Supplementary file 1 — Additional file 1. [file 12885_2021_7830_MOESM1_ESM.docx]

**Supplementary Table 1** Correlation between expression of miR-545 and the clinical characteristics of OC patients

| Characteristics | | N = 60 | miR-545 expression | | p value |
| --- | --- | --- | --- | --- | --- |
|  |  |  | Low (n = 29) | High (n =31) |  |
| Age (year) | ≥ 55 | 39 | 18 | 21 | 0.7876 |
|  | < 55 | 21 | 11 | 10 |  |
| CA125 (U/mL) | ≥ 60 | 42 | 22 | 20 | 0.4051 |
|  | < 60 | 18 | 7 | 11 |  |
| pTNM Stage | Ⅰ-Ⅱ | 28 | 8 | 20 | **0.0052 |
|  | Ⅲ-Ⅳ | 32 | 21 | 11 |  |

Note: OC, ovarian cancer; pTNM, pathologic tumor-node metastasis. Clinical characteristics of patients were analyzed using Fisher’s exact test; ***p* < 0.01.
